# Datasets of mung bean proteins and metabolites from four different cultivars

**DOI:** 10.1016/j.dib.2017.06.051

**Published:** 2017-07-04

**Authors:** Akiko Hashiguchi, Wei Zhu, Jingkui Tian, Setsuko Komatsu

**Affiliations:** a Faculty of Medicine, University of Tsukuba, Tsukuba 305-8577, Japan; b College of Biomedical Engineering & Instrument Science, Zhejiang University, Hangzhou 310027, China; c National Institute of Crop Science, National Agriculture and Food Research Organization, Tsukuba 305-8518, Japan; d Faculty of Life and Environmental Sciences, University of Tsukuba, Tsukuba 305-8572, Japan

**Keywords:** LC, liquid chromatography, MS, mass spectrometry, GC, gas chromatograph, TOF, time-of-flight, Mung bean, Health-promoting food, Nutritional/nutraceutical value, Metabolic network, Metabolomics, Proteomics

## Abstract

Plants produce a wide array of nutrients that exert synergistic interaction among whole combinations of nutrients. Therefore comprehensive nutrient profiling is required to evaluate their nutritional/nutraceutical value and health promoting effect. In order to obtain such datasets for mung bean, which is known as a medicinal plant with heat alleviating effect, proteomic and metabolomic analyses were performed using four cultivars from China, Thailand, and Myanmar. In total, 449 proteins and 210 metabolic compounds were identified in seed coat; whereas 480 proteins and 217 metabolic compounds were detected in seed flesh, establishing the first comprehensive dataset of mung bean for nutraceutical evaluation.

**Specifications Table**

**Table:** 

Subject area	Biology
More specific subject area	Plant Science
Type of data	Figure, Tables
How data was acquired	Gel-free/label-free proteomic analysis using data-dependent acquisition mode on a LTQ Orbitrap mass spectrometer coupled to an Ultimate 3000 nanoLC system.
	Metabolic analysis using an Agilent 7890 GC system coupled with a Pegasus HT TOF-MS.
Data format	Filtered
Experimental factors	Comparison of metabolic pathways in seed coat and flesh of mature mung bean
Experimental features	Whole-protein and metabolic compounds of mung bean coat and flesh were identified from four cultivars.
Data accessibility	Datasets are directly provided with this article.

**Value of the data**

● The data represent comprehensive repository of protein and primary metabolites contained in seed coat and flesh of mung bean, a medicinal plant with heat alleviating activity.
● This experimental design allows multiomics analysis of metabolic pathways of seed coat and flesh of mung bean.
● Differences in metabolic pathways and bioactive compound-containing pattern between seed coat and flesh were revealed using this data as described in [1].

## 1 Data

**Fig. 1:**
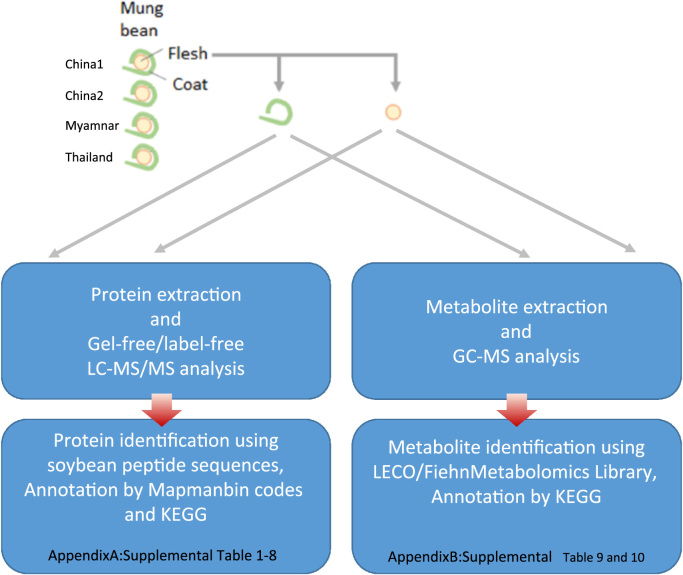
Flowchart of experiments and data processing.

The data here represent different omics approaches to understand the mung bean metabolic pathways and compound-containing pattern in seed coat and flesh. The dataset is associated with the research article in BBA Proteins and Proteomes entitled “Proteomics and metabolomics-driven pathway reconstruction of mung bean for nutraceutical evaluation” and contains eight lists of proteins and two lists of metabolites obtained from four cultivars originated from different habitats (Fig. 1).

## 2 Experimental design, materials and methods

### 2.1 Plant materials

Mung beans (Vigna radiata (L.) R. Wilczek) from different habitats in Asian countries were purchased from local supermarkets in Tokyo and Yokohama, Japan. These cultivars were referred as China 1, China 2, Thailand, Myanmar, respectively, according to their habitats. For protein and metabolite extraction, mung bean seeds was soaked in milliQ water to separate coat and flesh.

### 2.2 Gel-free/label-free proteomic analysis

The coat and flesh was ground to powder in liquid nitrogen using a mortar and pestle and transferred to an acetone solution containing 10% trichloroacetic acid and 0.07% 2-mercaptoethanol. The proteins were extracted as described in [1]. After enrichment with methanol and chloroform to remove any detergent, proteins were digested into tryptic peptides with trypsin and lysyl endopeptidase (Wako, Osaka, Japan). Peptide identification was performed by nanoliquid chromatography (LC) MS/MS with a nanospray LTQ Orbitrap mass spectrometer (Thermo Fisher Scientific, San Jose, CA, USA) coupled to an Ultimate 3000 nanoLC system (Dionex). Full-scan mass spectra were acquired in the mass spectrometer over 400–1500 m/z with a resolution of 30,000 in a data dependent mode as previously described [2], [3]. Proteins were identified by Mascot search engine (version 2.5.1, Matrix Science, London, UK) of a soybean peptide database (55,787 sequences) constructed from the Phytozome (version 9.1; http://www.phytozome.net/soybean). The acquired raw data files were processed by Proteome Discoverer software (version 1.4.0.0288; Thermo Fisher Scientific). Peptides with a percolator ion score of more than 13 (*p*<0.05) alone were used for analysis. Proteins with single matched peptide were also taken into consideration. Protein abundance in mol% was calculated based on the emPAI value [4]. Appendix file A contained the datasets with identified proteins from seed coat and flesh of four different cultivars. In total, 449 and 480 proteins were identified in seed coat and flesh, respectively, as described in [1].

### 2.3 Metabolomic analysis

Metabolites were extracted from dried sample powder with 0.4 mL extraction liquid (V methanol:V water=3: 1) using ball mill (JXFSTPRP-24, Shanghai Superscience Technology, Shanghai, China) as described in [1]. Gas chromatograph time-of-flight (GC TOF)-MS analysis was performed according to [5] using an Agilent 7890 GC system (Agilent, Palo Alto, CA, USA) coupled with a Pegasus HT TOF-MS (LECO, St Joseph, MI, USA). Peak analysis was performed by Chroma TOF 4.3X software (LECO) and LECO-Fiehn Rtx5 [6]. The similarity value obtained from the LECO/Fiehn Metabolomics Library was used for the evaluation of the accuracy of the discriminating compound identification is reliable. If the similarity is less than 200, the compound is defined as an “analyte”. The compound with a similarity between 200 and 700 is considered as a putative annotation. Appendix file B contains identified metabolic compounds from seed coat and flesh of four different cultivars.

### 2.4 Annotation of proteins and metabolites with KEGG identifiers

Proteins were categorized based on function using MapMan bin codes [7]. For pathway mapping, identifiers in the Kyoto Encyclopedia of Genes and Genomes (KEGG) database [7] (http://www.genome.jp/kegg/) were retrieved from MapMan system or KEGG COMPOUND search.
