# Supplementary figures and images for: Datasets of mung bean proteins and metabolites from four different cultivars
Source: Data Brief. 2017 Jul 4;13:703–6. doi: 10.1016/j.dib.2017.06.051 (PMC5510489; doi:10.1016/j.dib.2017.06.051)

Figure 1

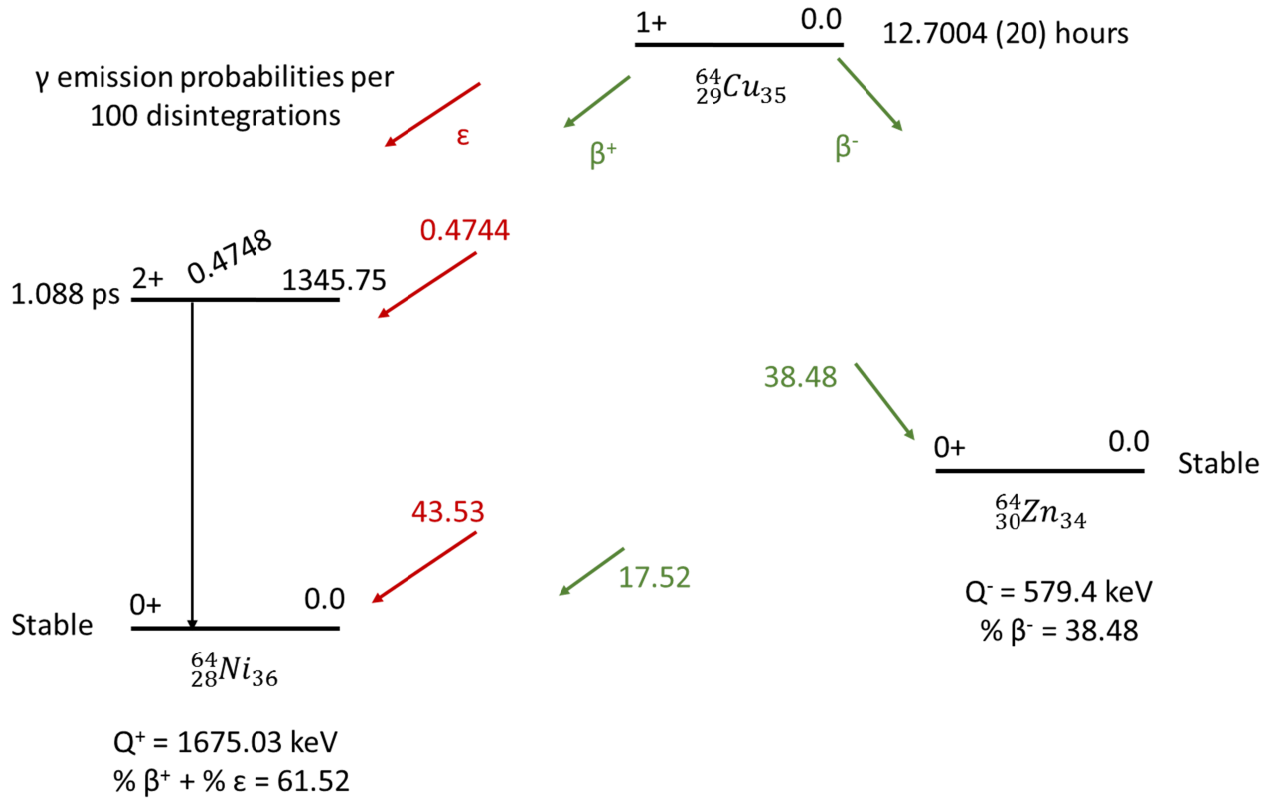

Figure 2

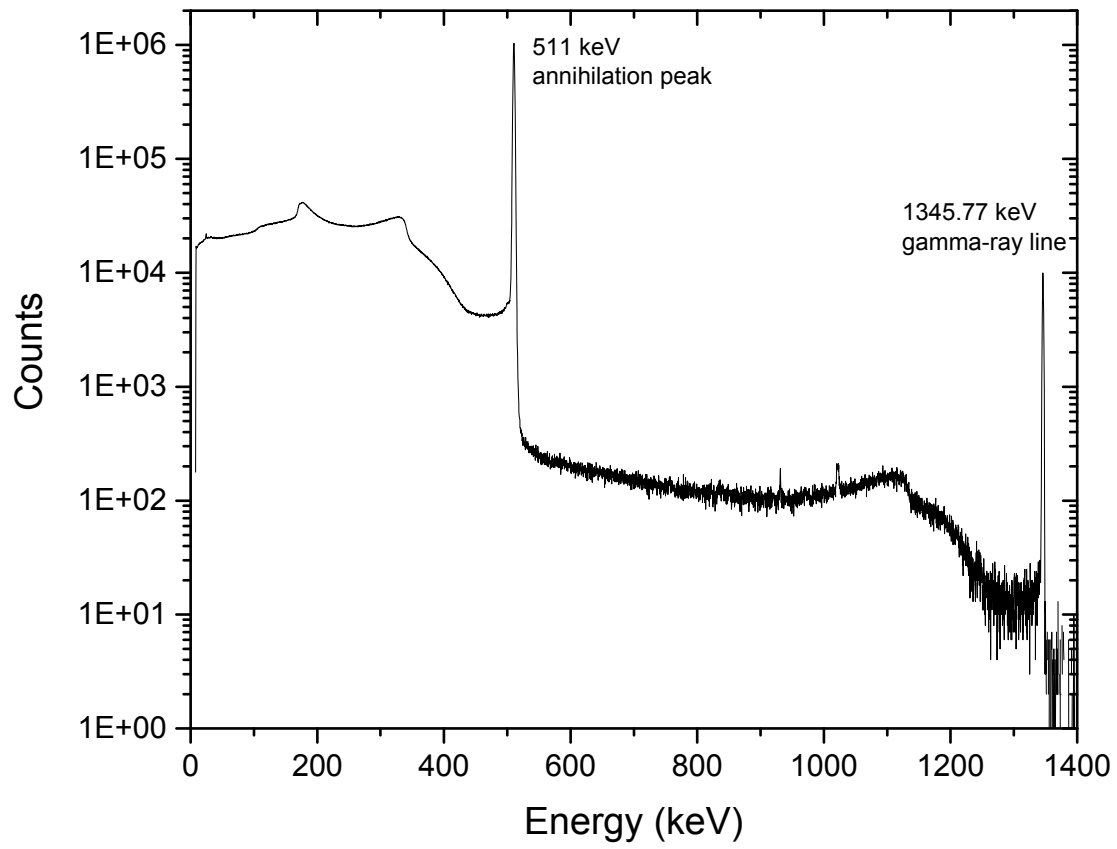

Figure 3

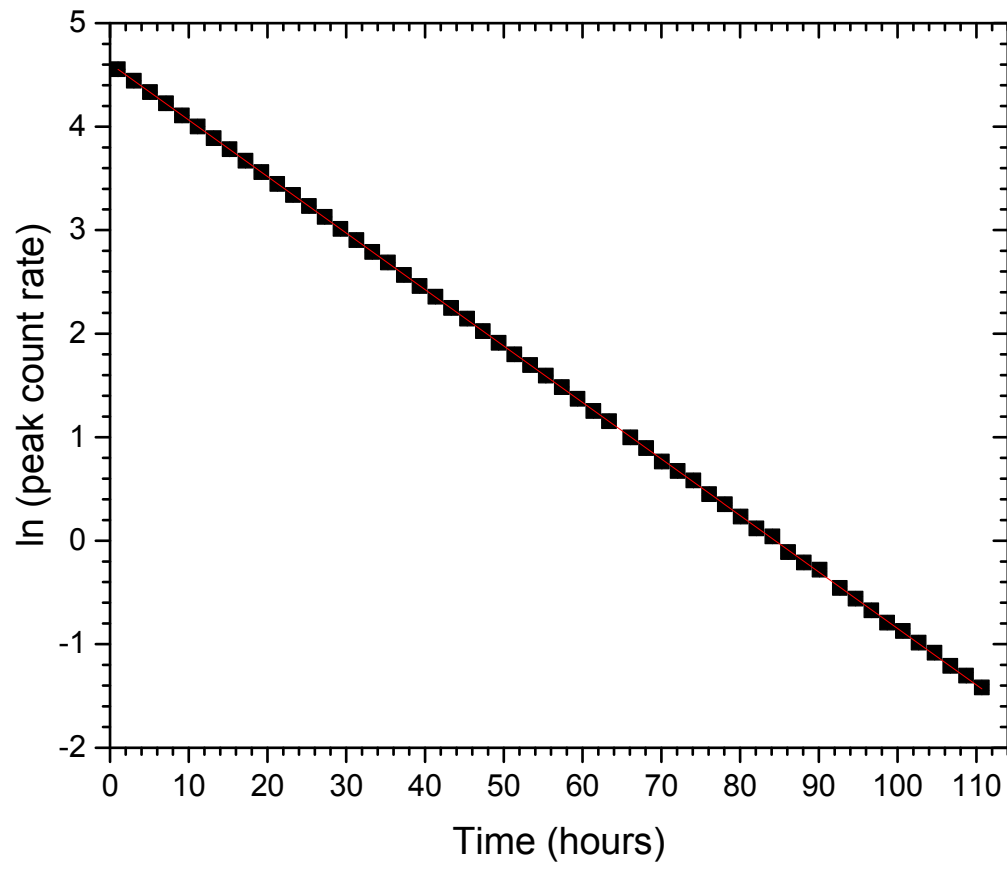

Figure 4

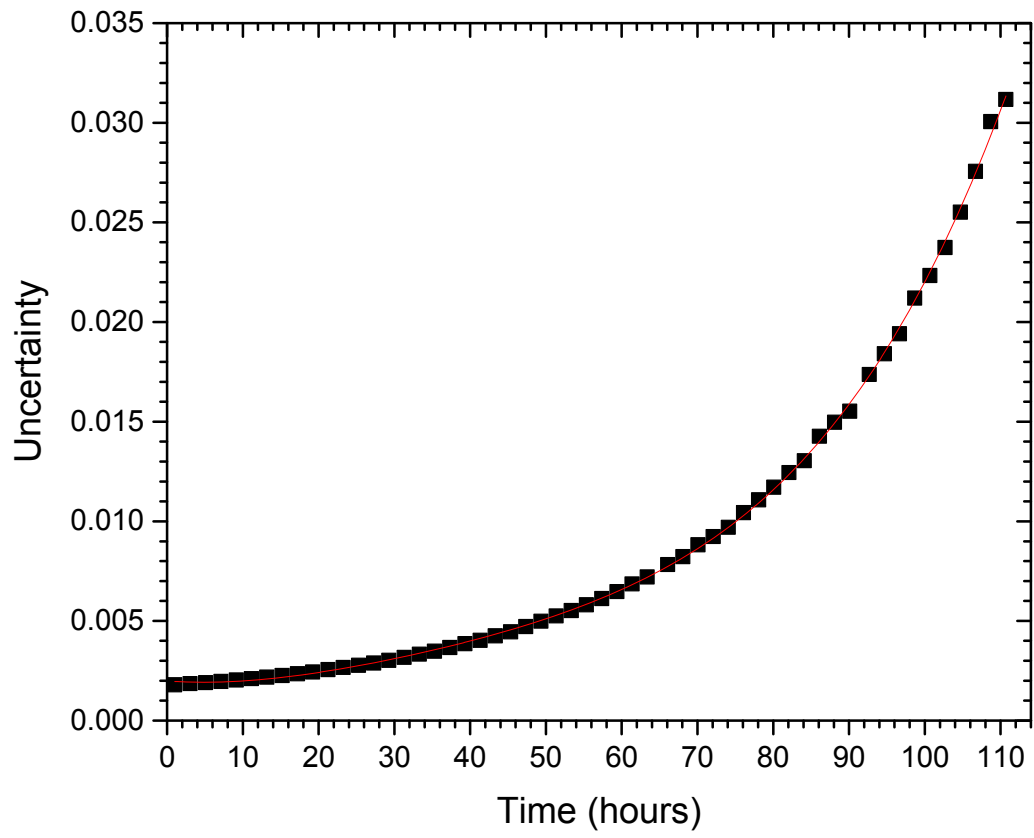

Figure 5

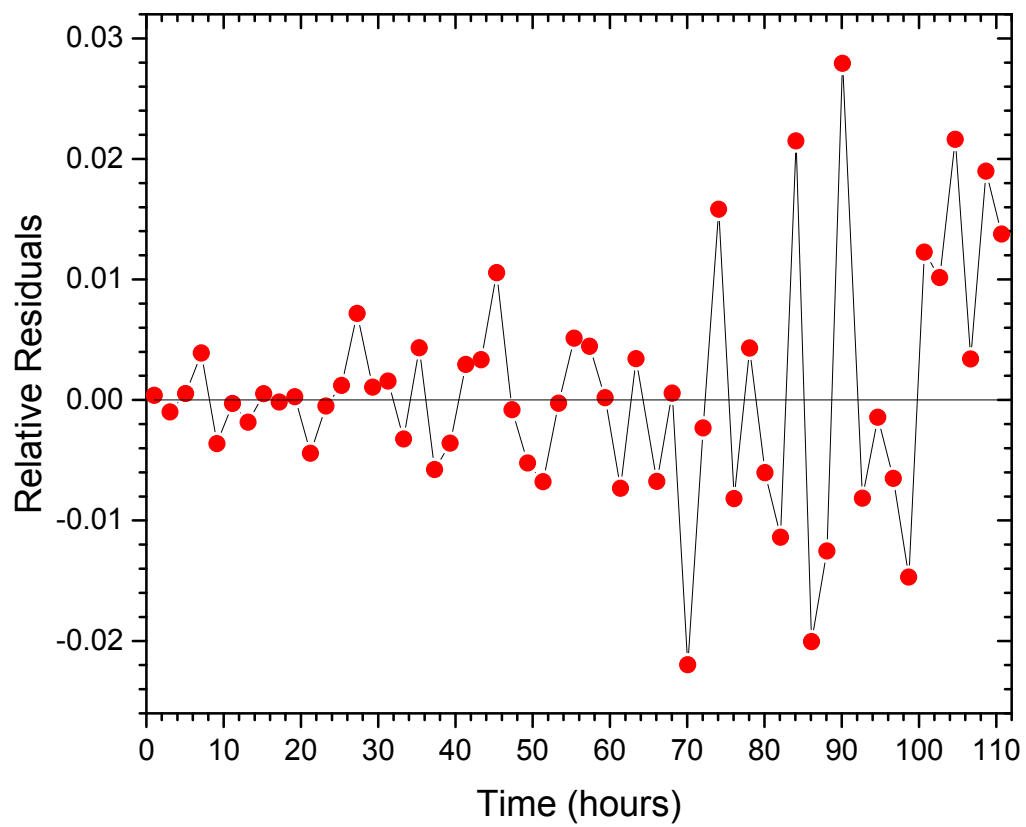

Figure 6

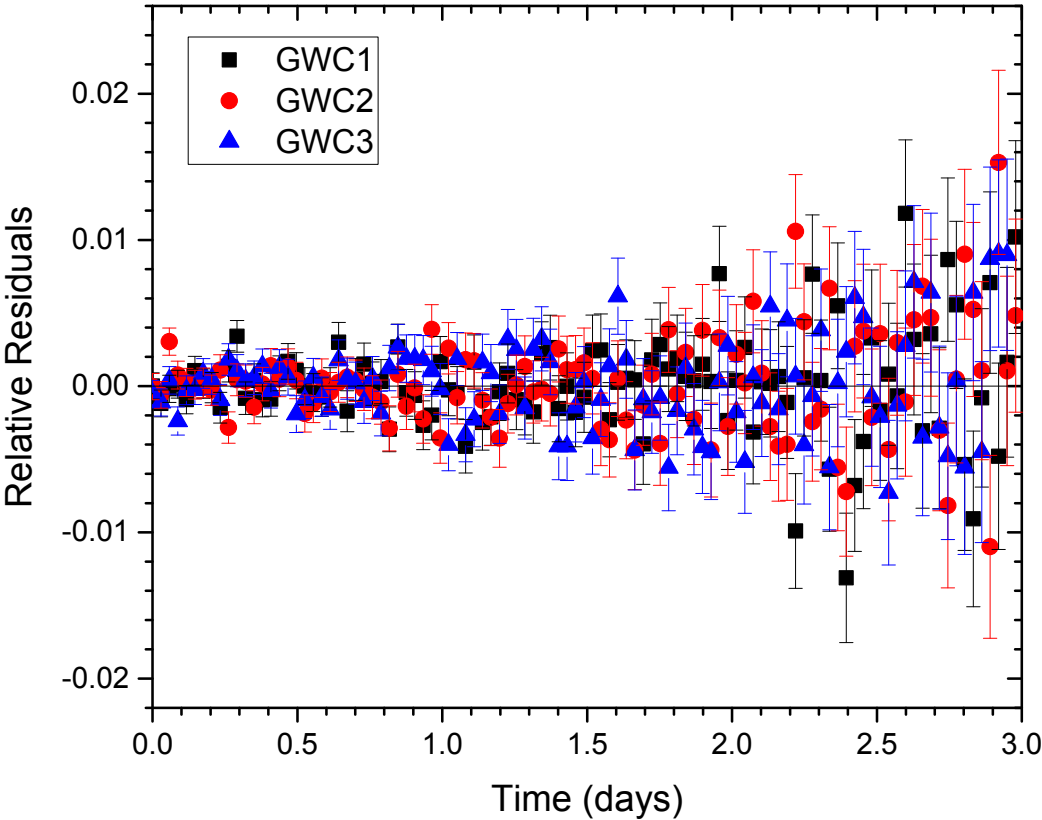

Figure 7

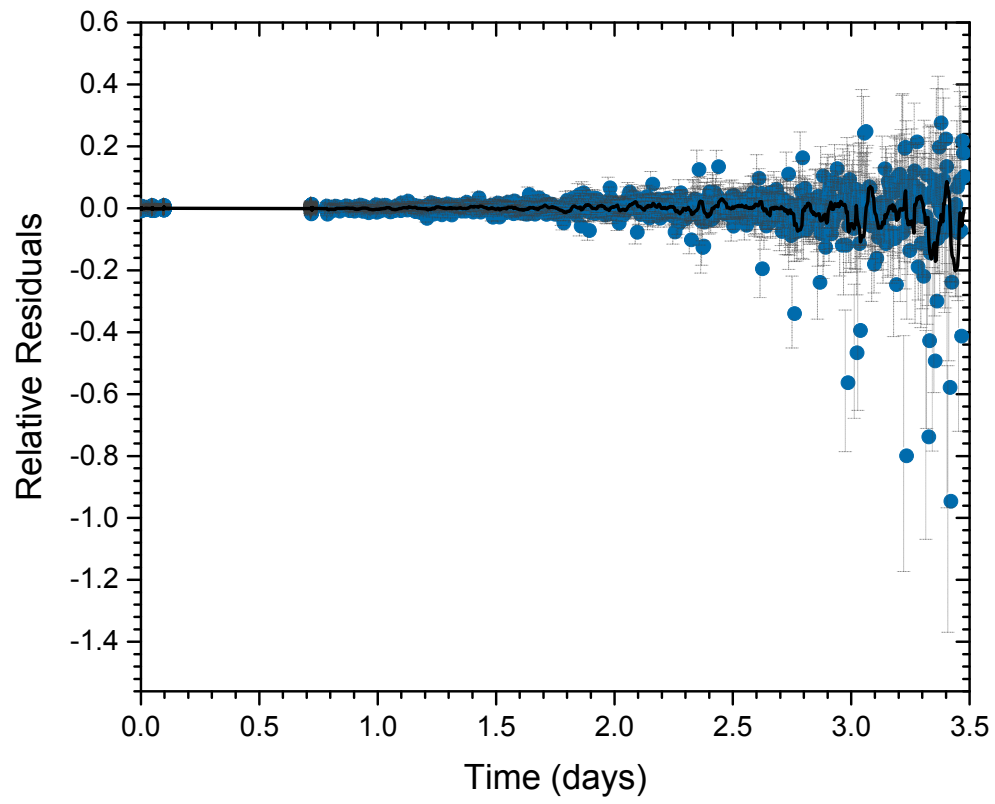

Figure 8

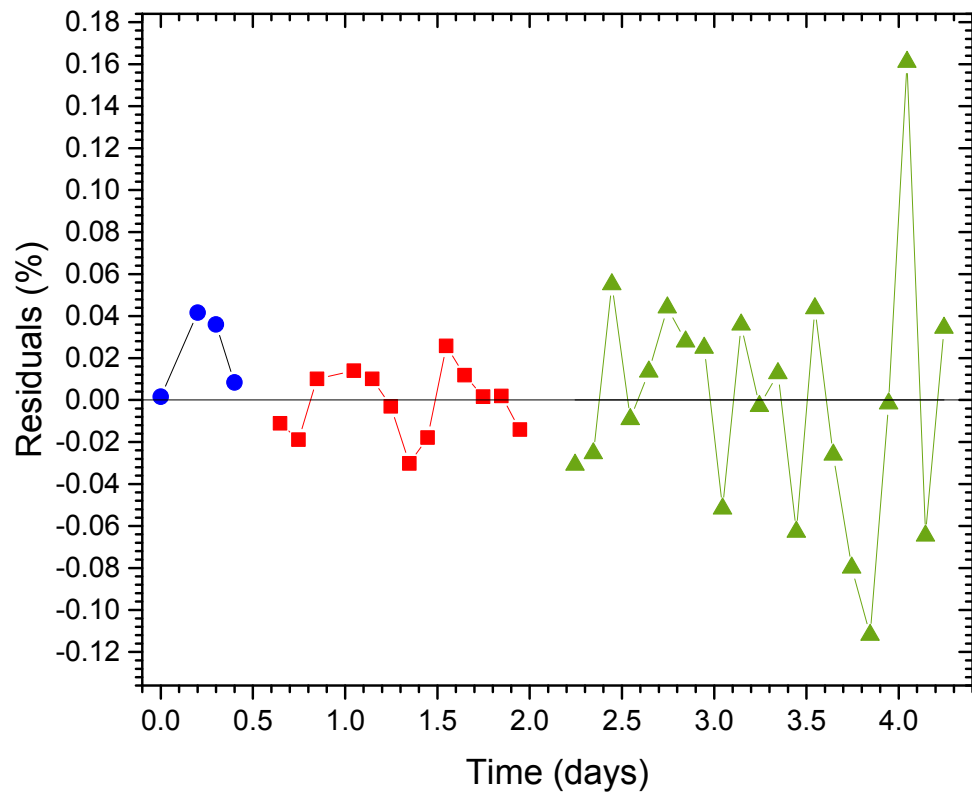

Figure 9

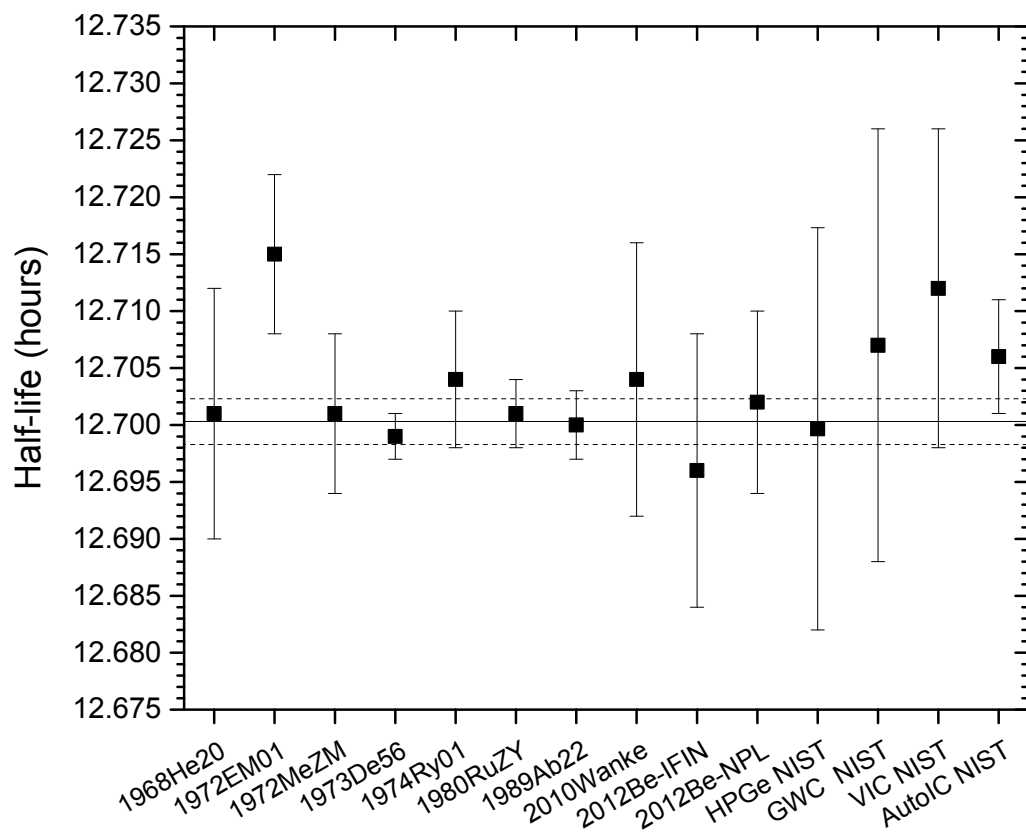

Supplement: Supplementary file 2 — Supplementary material [file mmc2.pdf]
